# Feasibility of gadolinium contrast use in transcatheter aortic valve implantation: a non-iodine-based procedure—case report

**DOI:** 10.1093/ehjcr/ytaf585

**Published:** 2025-11-13

**Authors:** Omar A Oliva, Jerome Van Rothem

**Affiliations:** Interventional Cardiology Department, Clinique Pasteur, 45, Avenue de Lombez, Toulouse 31000, France; Interventional Cardiology Department, Clinique Pasteur, 45, Avenue de Lombez, Toulouse 31000, France

**Keywords:** Adverse drug reaction, Aortic valve replacement, Aortic stenosis, Case report, Generalized bullous fixed drug eruption

## Abstract

**Background:**

Iodine-based contrast agents can be associated to uncommon side effects, as the generalized bullous fixed drug eruption (gbFDE), that cannot be counteracted by drugs.

**Case summary:**

We report a case of TAVI performed with a gadolinium-based contrast agent in a patient that had previously experienced a severe form of gbFDE. The patient was successfully treated by a balloon-expandable prosthesis implanted under the guidance of a total of 30 cc of gadolinium injections, avoiding any side effect.

**Discussion:**

Thus, the planning of TAVI procedure with zero iodinated contrast is mandatory in high-risk patients for such complication and it is feasible, safe with a gadolinium-based contrast.

Learning points

- Transcatheter aortic valve implantation can be performed without iodinated contrast in patients with absolute contraindications, as generalized bullous fixed drug eruption (gbFDE).
- Gadolinium-based contrast agents may serve as a safe and effective alternative when used in low volume and with appropriate dilution, maintaining a good image quality and avoiding the risk of ventricular arrhythmias.
- Careful pre-procedural planning, including non-contrast imaging and choice of prosthesis, is essential.

## Introduction

Transcatheter aortic valve implantation (TAVI) is an established and effective treatment for aortic valve stenosis.^1^ The standard procedure involves the use iodine-based contrast, particularly during the phase of implantation.

Iodine-based contrast agents can be associated with side effects, as allergic reactions, which need specific and prompt treatment, before or after their injection, depending on whether the allergy is known. However, there is the possibility of other, less common side effects, as the generalized bullous fixed drug eruption (gbFDE), which usually are associated with medication, as antibiotics and nonsteroidal anti-inflammatory drugs.^2,3^ Thus, the planning of TAVI procedure can become challenging, since there are no proven treatments for the prevention of these syndromes.

Herein we present a case of TAVI on a native valve, which was performed without iodinated contrast, thanks to careful pre-procedural planning in a patient that had previously experienced a severe form of gbFDE.

## Summary figure

Angiographic evidence of the gadolinium injections and risks/benefits list.

**Figure:**
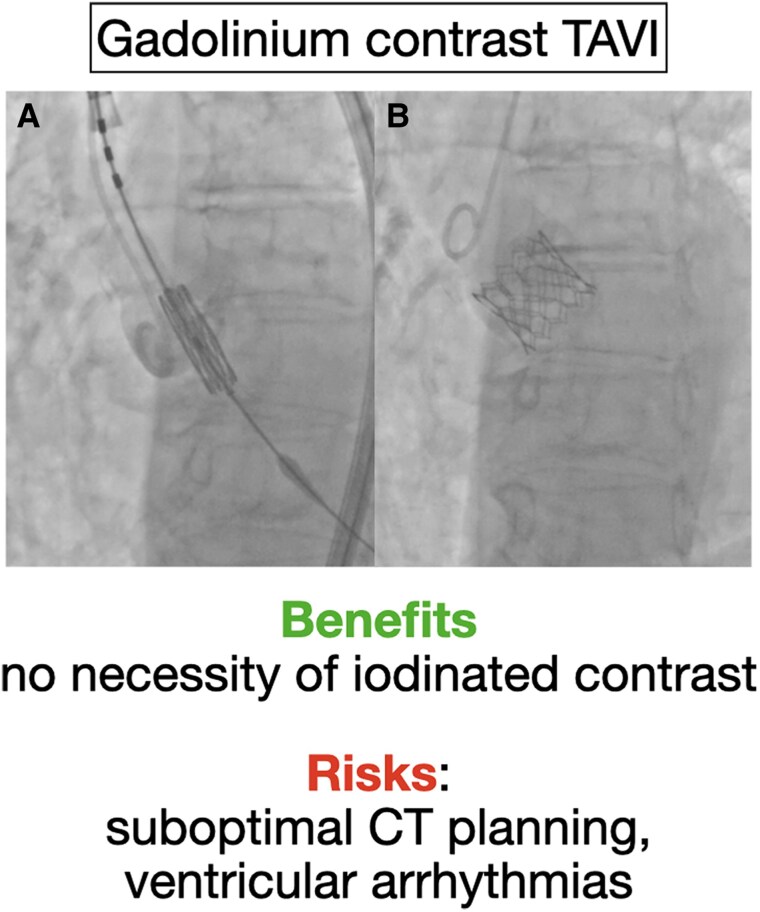


## Case presentation

A 72-year-old woman was admitted to our intensive care unit for an episode of acute heart failure in 2023. Transthoracic echocardiography showed a low flow low gradient severe aortic stenosis with depressed ejection fraction (35%) associated with severe mitral regurgitation. In 2020 she had experienced a severe form of gbFDE involving 70% of the body surface area after a diagnostic coronary angiography that was linked to the iodine-based contrast use, confirmed by the biopsy and allergologic tests. The patient had a history of breast cancer treated with radiotherapy in addition to the poor clinical status, thus TAVI was proposed. However, she was still shocked by the adverse event connected to the coronary angiography and since the only preventive measure is to avoid a new iodine injection,^2^ she categorically refused a TAVI procedure and was discharged on increased diuretic treatment. The patient’s clinical status declined further and she was again admitted for a second episode of acute heart failure. She was reevaluated and a zero iodinated-contrast strategy was proposed: the pre-TAVI computed tomography (CT) was performed without contrast injection (the patient had a high calcium burden, allowing the anulus measurement—22.9 mm perimeter-derived diameter- and vascular access evaluation) and a gadolinium-based contrast was chosen for the procedure, in order to avoid any crossed-reaction.

The procedure was performed under local anaesthesia and fluoroscopic guidance. A 23 mm balloon-expandable Sapien 3 valve was implanted with the help of two 15 cc injection of gadolinium to visualize the aortic root, to reach the correct implantation depth (Summary *Figure A* and Supplementary material online, *Video S1*), and to assess the correct implantation depth (Summary *Figure B* and Supplementary material online, *Video S2*). No transvalvular gradient or paravalvular leak were shown by the haemodynamic evaluation. The patient did not experience any form of gbFDE and was discharged at Day 6 due to temporary conduction abnormalities. At 6-month follow-up, the patient had an optimal clinical status (New York Heart Association functional Class I) without ECG conduction disturbances, improvement in the ejection fraction (58%) and reduction in mitral regurgitation, associated with a satisfying device performance: 12 mmHg of transprosthetic mean gradient without paravalvular leak.

## Discussion

During the planning of TAVI procedures, the risk of iodinated-based contrast agents should be evaluated, especially in patients with chronic kidney disease or history of severe side effects. In the reported case, several technical arrangements were made: firstly, a zero-contrast CT scan was performed; secondly, a balloon expandable prosthesis was chosen since it could allow a fast deployment and finally, the use of gadolinium to identify the correct annular plane. There are a few studies that showed the safety and efficacy of gadolinium during coronary angiographies in patients with a strict contraindication to iodinated products^4^; however, to our knowledge, this is not the first case that it has been employed in TAVI. Nagarja *et al*. and Alshehri *et al*. performed a gadolinium-based TAVI to avoid an iode allergic reaction. The latter, used a combination of non-contrast cardiac CT, transoesophageal echocardiogram and femoral Doppler ultrasound for procedural planning.^5,6^ However, this is the first case where it was absolutely contraindicated since there are no available treatments for gdFDE, as cortisone premedication is ineffective.

Due to the risk of developing ventricular arrhythmias during coronary angiographies (3.8%),^4^ we diluted the gadolinium 1:1 with sodium chloride and the number of injections was limited to two. In fact, Juneman *et al*.^7^ suggest that ventricular fibrillation can occur due to the combination of pressure damping and hyperosmolar gadolinium contrast. Therefore the dilution associated to a non-selective injection in the aorta was deemed sufficient to avoid arrhythmias. An alternative strategy could have been the usage of the pigtail catheter as a marker of the aortic annulus, however, its positioning at the nadir of the non-coronary cusp is not always reliable and a confirmation by a contrast injection is wiser. Another alternative method could have been intraprocedural transoesophageal echocardiography, however it is less tolerated by the patient and it could require the necessity of general anaesthesia.

Our case report confirms the feasibility, the safety, and the image quality of a gadolinium-based contrast imaging for TAVI. Further studies are warranted to validate these findings.

Written informed consent was obtained from the patient for the publication of this case report. The authors confirm that this work complies with the ethical standards of the Committee on Publication Ethics and that all appropriate patient privacy and confidentiality measures have been observed.

The data of the current study are available from the corresponding author, OAO, upon reasonable request, due to privacy/ethical constraints.

## Lead author biography


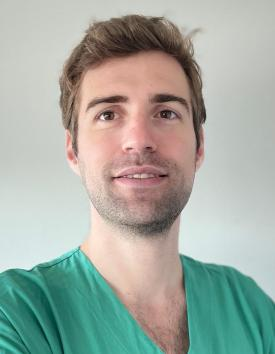


I am a cardiologist specialized in coronary and structural interventions. I am pursuing a fellowship at the Clinique Pasteur, Toulouse. Our aim is to bring innovation and to share new techniques.

## Supplementary Material

ytaf585_Supplementary_Data

## Data Availability

Data can be asked to the corresponding author.

## Supplementary material

**Funding:** This study was supported by internal institutional funds.

## References

[1] Vahanian A, Beyersdorf F, Praz F, Milojevic M, Baldus S, Bauersachs J, et al 2021 ESC/EACTS guidelines for the management of valvular heart disease. Eur Heart J 2022;43:561–632. 34453165 10.1093/eurheartj/ehab395

[2] Patel S, John AM, Handler MZ, Schwartz RA. Fixed drug eruptions: an update, emphasizing the potentially lethal generalized bullous fixed drug eruption. Am J Clin Dermatol 2020;21:393–399. 32002848 10.1007/s40257-020-00505-3

[3] Gavin M, Sharp L, Walker K, Behrens E, Akin R, Stetson CL. Contrast-induced generalized bullous fixed drug eruption resembling stevens-johnson syndrome. Proc (Bayl Univ Med Cent) 2019;32:601–602. 31656435 10.1080/08998280.2019.1644147 PMC6794010

[4] Guragai N, Roman S, Vasudev R, Rampal U, Randhawa P, Shamoon F, et al Gadolinium-based coronary angiography in a patient with prior known anaphylaxis to iodine-based dye. J Community Hosp Intern Med Perspect 2021;11:286–288. 33889340 10.1080/20009666.2021.1890337 PMC8043562

[5] Nagaraja V, Gulati R, Alkhouli MA, Eleid MF, Williamson EE, Rihal CS. First transcatheter aortic valve replacement with gadobutrol in a patient with severe contrast allergy. Cardiovasc Revasc Med 2022;40:123–125. 35246411 10.1016/j.carrev.2022.02.021

[6] Alshehri B, Alamri H, Alghasab N, Ahmed J, Alshehri F, Samargandy S, et al Gadolinium-guided transcatheter aortic valve implantation in a patient with renal impairment and a history of severe allergic reaction to iodinated contrast Media. Interv Cardiol 2025;20:e03. 40028271 10.15420/icr.2024.15 PMC11865668

[7] Juneman E, Saleh L, Thai H, Goldman S, Movahed MR. Successful coronary angiography with adequate image acquisition using a combination of gadolinium and a power injector in a patient with severe iodine contrast allergy. Exp Clin Cardiol 2012;17:17. 23204895 PMC3383362

